# Supplementary material for: Efficacy and safety of short-term therapy with indigo naturalis for ulcerative colitis: An investigator-initiated multicenter double-blind clinical trial
Source: PLoS One. 2020 Nov 5;15(11):e0241337. doi: 10.1371/journal.pone.0241337 (PMC7644062; doi:10.1371/journal.pone.0241337)
Supplement: S1 Table — (DOCX) [file pone.0241337.s001.docx]

**S1 Table: Comparison between marked responders and nonmarked responders.**

|  | Marked response | Nonmarked response | p-value |
| --- | --- | --- | --- |
|  | (n=14) | (n=9) |  |
| Age | 39.6±16.3 | 47.0±20.4 | 0.35 |
| Height | 162.3±10.0 | 162.2±6.13 | 0.98 |
| Body weight | 52.5±10.0 | 59.1±11.1 | 0.15 |
| Lichtiger index | 9.14±2.03 | 8.89±1.83 | 0.77 |
| WBCs | 5421.0±2623.3 | 6543.5±3282.0 | 0.37 |
| Hb | 13.5±1.41 | 13.2±1.61 | 0.58 |
| Plt | 29.3±10.0 | 34.2±10.9 | 0.28 |
| AST | 17.4±5.59 | 16.6±5.68 | 0.72 |
| ALT | 13.8±8.66 | 12.1±5.82 | 0.62 |
| UN | 10.9±1.81 | 8.84±3.84 | 0.10 |
| Cr | 0.66±0.14 | 0.77±0.20 | 0.15 |
| TP | 7.05±0.39 | 6.99±0.55 | 0.75 |
| Alb | 4.04±0.40 | 3.93±0.56 | 0.60 |
| CRP | 0.19±0.14 | 0.66±1.15 | 0.26 |

WBCs: white blood cells; Hb: hemoglobin; Plt: platelets; AST: aspartate aminotransferase; ALT: alanine aminotransferase; UN: urea nitrogen; Cr: creatinine; TP: total protein; Alb: albumin; CRP: C-reactive protein; NS: Not significant
