# Supplementary material for: Efficacy and safety of short-term therapy with indigo naturalis for ulcerative colitis: An investigator-initiated multicenter double-blind clinical trial
Source: PLoS One. 2020 Nov 5;15(11):e0241337. doi: 10.1371/journal.pone.0241337 (PMC7644062; doi:10.1371/journal.pone.0241337)
Supplement: S1 File — (DOCX) [file pone.0241337.s002.docx]

潰瘍性大腸炎に対する

青黛の有効性と安全性の評価

多施設共同プラセボ対照二重盲検試験

試験計画書

Ver.1-11

研究代表・実施責任者：

東京慈恵会医科大学附属柏病院　消化器・肝臓内科　内山　幹

協力者：

東京慈恵会医科大学附属　柏病院　消化器・肝臓内科　大草敏史

東京慈恵会医科大学附属柏病院　消化器・肝臓内科　小田原俊一

東京慈恵会医科大学附属柏病院　消化器・肝臓内科　伊藤善翔

東京慈恵会医科大学附属柏病院　消化器・肝臓内科　高見信一郎

臨床試験実施予定期間：倫理審査会承認後～2019年3月

目次

1. 試験の背景と目的
2. 対象患者
3. 被験者に説明し同意を得る方法
4. 試験の方法および評価項目
5. 選択基準
6. 除外基準
7. プロトコール中止基準
8. 試験実施期間
9. 重篤な有害事象発生時の取り扱い
10. その他の有害事象の評価と報告
11. 試験の終了と早期中止
12. 被験者の人権（個人情報の保護）に対する配慮
13. 臨床研究保険
14. 予想される医学上の貢献、および本研究課題の出口
15. 研究に参加した場合に被験者が受ける不利益
16. 患者の費用負担
17. 研究資金および利益相反
18. 人を対象とする医学系研究に関する倫理指針，ヘルシンキ宣言への対応
19. 臨床研究計画の登録
20. 研究組織
21. 研究事務局
22. 実施計画書等の変更
23. 連絡先または相談窓口

**１．研究の背景と目的**

潰瘍性大腸炎（Ulcerative colitis、以下UC）は食生活の欧米化に伴い急速に増加している難治性炎症性腸疾患である。従来、UCに対しては５－アミノサリチル酸（5-ASA）製剤やステロイド、免疫調整薬（アザチオプリン、6-MP）などの薬剤や血球成分吸着除去療法（L-CAP、G-CAP）が用いられていたが、近年、抗TNF-α抗体薬（インフリキシマブ、アダリムマブ）やカルシニューリン阻害薬（タクロリムス）が登場し、治療法の選択が多岐にわたる。その一方、ある種の健康食品も患者内で広まっているが、その実態は定かではない。

UCの治療は便回数や血便などの臨床症状による疾患活動性指標(Lichtiger index)や内視鏡による活動性評価(Mayoサブスコア)を基に治療法の調整が行われている。潰瘍性大腸炎治療指針（2014年3月改訂、『難治性腸管障害に関する調査研究』班（渡辺斑）、平成25年度分担研究　文献１）では5-ASA製剤を基本薬とし、不応例にはステロイド、血球成分吸着除去療法、免疫調整薬など様々な治療選択肢があげられており、治療の標準化は行われていない。こうした治療にまぎれて、健康食品が使用されているものと考えられるが、それらが経過に及ぼす影響に関する報告はない。

青黛はキツネノマゴ科のリュウキュウアイ、マメ科のタイワンマツナギ、アブラナ科の植物のホソバタイセイなどの葉や茎に含まれる青色色素で、日本では健康食品として入手が可能となっている。一方、青黛を主成分とした漢方薬シレイサンの浣腸は中国では潰瘍性大腸炎の治療に用いられ，日本国内でもプラセボを対象とした坐剤の有効性が報告されている^１^．また近年、複数例のUC患者に青黛の経口投与が奏効したことも報告されている^2^が、限られた症例報告であるため、科学的なエビデンスに乏しい。

本研究では、青黛の有効性と安全性をより高い科学的エビデンスでの結論を得るため，多施設共同プラセボ対照二重盲検試験にて評価する．

**２．対象患者**

各施設Inflammatory bowel disease(IBD)外来に通院加療中のUC患者で，既存治療に不耐または不応の活動期軽～中等症の患者計68例^＊^．UCは小児・若年者に好発する疾患であるため，本研究では未成年を含む16歳からを対象とする．16歳以上，20歳未満の未成年者は，本人ならびに保護者の同意を得ることとし，小児例 ( 16歳未満 ) ，代諾者として保護者あるいは本人から同意が得られない16歳以上20歳未満の未成年患者症例は対象外とする．

＊青黛群の有効率を70%、プラセボ群の有効率を30%、αエラー 0.05、Power 0.8とすると必要症例数は各29例の計58例となる。脱落を各群5例と見積もり各34例の合計68例とした。

**３. 被験者に説明し、同意を得る方法**

**３．１　同意の取得**

臨床研究責任医師または分担医師は、本研究の実施に先立ち、以下の事項が含まれている同意説明文書を用い十分に説明し、患者が内容をよく理解したことを確認した上で本研究への協力について患者本人の自由意思による同意を所定の同意文書にて得る。その際、患者が質問する機会と研究に協力するか否かを判断するのに十分な時間を与える。同意文書には説明を行った試験責任医師または分担医師、患者本人が署名し、各自日付を記入する。患者が未成年の場合は、原則として母親もしくは父親を代諾者として選定してインフォームド・コンセントを得る。ただし患者・対照が16歳以上20未満の場合は、上記代諾者に加え、本人の同意も得る。

1. 本臨床試験への参加は任意であり、本試験に参加しない場合でも不利益を受けないこと。そして同意した場合でも随時これを撤回できることについて。
2. 試験計画の目的について
3. 試験の方法について
4. 試験への予定参加期間について
5. 試験に参加する予定の患者数について
6. 試験に伴う検査について
7. 予想される効果および副作用、臨床検査値異常について
8. 他に取り得る治療法について

９） 個人プライバシーが守られた上で、本試験の成果が公表される可能性があること

１０） 本試験での治療に係わる費用の負担について

１１） 健康被害発生時の補償について

１２） あらたな情報が出たときには直ちに情報が知らされることについて

１３） 試験中並びに終了後のアンケート、データの取り扱いについて

１４） 試験に関する問い合わせ及び連絡先について

**３．２　同意の取得時期**

文書による同意の取得時期は登録前とする．

**４．試験の方法および評価項目**

**４．１ ランダム化と症例登録**

　　症例登録は臨床研究支援システムHOPE eACReSSを用いる。このシステムはWeb上で患者登録とランダム化が可能である．このシステムを用いて青黛群とプラセボ群に無作為に群別を行う．患者情報、投与前後の疾患活動性指標(Lichtiger index)、内視鏡所見、検査結果、副作用の有無とその内容が入力できる。

＜Lichtiger Index＞

| **Symptom** | **Score** |
| --- | --- |
| *Diarrhea (no. of daily stools)* |  |
| 0–2 | 0 |
| 3 or 4 | 1 |
| 5 or 6 | 2 |
| 7–9 | 3 |
| 10 | 4 |
| *Nocturnal diarrhea* |  |
| No | 0 |
| Yes | 1 |
| *Visible blood* | (% of movements) |
| 0 | 0 |
| Less than 50 | 1 |
| Greater than 50 | 2 |
| 100 | 3 |
| *Fecal incontinence* |  |
| No | 0 |
| Yes | 1 |
| *Abdominal pain or cramping* |  |
| None | 0 |
| Mild | 1 |
| Moderate | 2 |
| Severe | 3 |
| *General well being* |  |
| Perfect | 0 |
| Very good | 1 |
| Good | 2 |
| Average | 3 |
| Poor | 4 |
| Terrible | 5 |
| *Abdominal tenderness* |  |
| None | 0 |
| Mild and localized | 1 |
| Mild to moderate and diffuse | 2 |
| Severe or rebound | 3 |
| *Need for antidiarrhea drugs* |  |
| No | 0 |
| Yes | 1 |

**４．２　製剤またはプラセボの作成・管理**

　　青黛およびプラセボはウチダ和漢薬にて各100mgを1カプセルへ充填する。プラセボはコメデンプンを使用する。青黛とプラセボは高温多湿を避け密閉容器で管理し、使用期限が過ぎたものは使用しない。これらは製剤Aまたは製剤Bとして1症例2週間分をプラスチックボトルに除湿剤とともにまとめ、各施設へ配送する。送付後の製剤も高温多湿を避けた状態で管理し、使用期限を過ぎたものは使用しないこととする。

　　割り当てられた製剤を患者に手渡す際には，プラスチックボトルのキャップに，Web登録上の登録番号を油性マジックで記載する。

**４．３　服用方法**

　　通常服用している薬剤(5-ASA製剤など)に加えて、青黛またはプラセボのカプセルを1回5カプセル（500mg）、1日2回を2週間服用する。

**４．４　服用アンケート**

　　被験者に服用アンケートを印刷して渡し、服用状況、便回数、便性状、出血の程度を記入してもらう。

**４．５　試験終了時**

　　2週間後に臨床所見、内視鏡所見、検査結果、有害事象を入力する。中止した場合はその理由も記入する。投与期間を終了した時点で，余った製剤は回収し、各施設で盲検解除まで保管する。

| 実施項目 | | 投与期間 | | 中止時 |
| --- | --- | --- | --- | --- |
|  |  | 投与開始日（前） | 2週間後**^＊＊^** |  |
| 選択・除外基準・同意取得 | | ● | － | － |
| 被験者背景・前治療状況等の調査 | | ● | － | － |
| 副作用の調査 | | － | ● | ● |
| 評  価 | 疾患活動性指標(Lichtiger index) | ● | ● | ● |
|  | 血液検査**^＊＊＊^** | ● | ● | ● |
|  | 内視鏡検査**^＊^** | （●）  (投与開始2週間以内) | （●）  (投与終了1週間以内) | － |
|  | 患者服薬日誌 | － | ● | ● |
|  | 糞便採取******** | （●） | （●） |  |

**＊**任意。浣腸による直腸のみの観察も可。生検は必須ではない。

**＊＊**患者が継続して服用を希望した際は、健康食品として自分で青黛を購入し，主治医に申告したうえで服用していただく。

**＊＊＊**採血検査項目は，血算，総蛋白，アルブミン，CRP，赤沈を必須とする．

**＊＊＊＊**内視鏡検査を施行する場合には，腸液採取での代用も可とする．

**４．６　主要評価項目(Primary Endpoint**)

　　投与2週後における疾患活動性指標(Lichtiger index)の50%以上の改善割合*

*ベースラインの50%以上のスコア減少を「改善」と定義する。

**４．７　副次評価項目(Secondary Endpoint)**

投与後の内視鏡所見(Endoscopic Mayo score)が1以上減少した被験者の割合**

**1以上のスコア減少を「改善」と定義する。

投与期間中に新たに身体的有害事象(頭痛、下痢、息切れ、発熱等)を来した被験者の割合

　　投与期間中に新たに臨床血液検査値異常を来した被験者の割合とその内容

　　投与前後の腸内細菌叢の変化

**４．８　盲検解除**

盲検解除は、緊急時に該当する症例について４．９の手順により行う場合を除いて，試験が終了し、データが固定された後に行う。

**４．９　エマージェンシーキーの管理・盲検解除手順**

エマージェンシーキーは割付けコード化担当者が保管し、症例の緊急時を除き開鍵しない。緊急時とは、有害事象又は重大な有害事象が発生し、割付けが青黛であるか否かにより対処法が異なる場合，および９章の重篤な有害事象の定義に該当する場合と定義する。

緊急事態が発生した場合は各施設の代表者にその旨を連絡し、研究代表者は割付け担当者(研究事務局; 筑波大学附属病院　鈴木英雄医師)に当該被験者番号と担当医師名を伝える。割付け担当者は当該症例についてのみ盲検解除を行い、当該症例の担当医師に割付けられた治療法を伝える。重篤な有害事象の定義に該当する場合は試験の継続の可否について研究代表者、及び試験責任医師で協議、決定する。

後治療方法を決定するための盲検解除は行わない。

**４．１０　統計解析の方法**

　　　青黛群とプラセボ群の2群において， Indexが50%以上改善した被験者の割合をChi-square testにて比較し，さらに上記した疾患活動指標の変化をWilcoxon検定にて評価する．これらの検定において青黛群の有効性が確認された場合には，罹患期間や病型などの各種患者背景項目を用いて多変量解析（Logistic回帰分析）を行い，有効予測因子の検討も行う．

**４．１１　モニタリングについて**

　　　モニタリングについては別途モニタリング計画書に定める．

**５．選択基準**

　・16歳以上

　・PS(ECOG)が0または1である。

・活動期軽～中等症の潰瘍性大腸炎(Lichtiger index 5-10)

・外来通院中である。

　・既存治療に不耐または不応である。（薬剤の投与量は問わない、減量中の再燃を含む）

・2週以内の血液検査で下記のすべての条件を満たす

i) ヘモグロビン≧9g/dl

ii) AST・ALT≦施設基準

iii) 血清クレアチニン≦施設基準

・試験参加に対して被験者から文書による同意が得られている。

**６．除外基準**

　・2週以内に5-ASA製剤(坐剤や注腸も含む)の開始、増量が行われている。

　・2週以内にステロイド(坐剤や注腸も含む)の開始、増量が行われている。

　・3か月以内に免疫調整剤(アザチオプリン、6-MP)の開始、増量が行われている。

　・3か月以内に血球成分除去療法が行われている。

　・3カ月以内に生物学的製剤(インフリキシマブ、アダリムマブ)またはタクロリムスの開始、増量が行われている。

　・青黛を含む漢方薬が使われたことがある。

　・妊娠中・妊娠の可能性のある、または授乳中の女性。

・精神病または精神症状を合併しており試験への参加が困難と思われる。

・活動性の細菌および真菌感染症を有する。（38.5℃以上の発熱を有し、画像診断もしくは細菌学的検査にて細菌感染が証明されている）

・3ヶ月以内に、心筋梗塞または不安定狭心症の既往を有する。

・コントロール不良の高血圧症を合併している。

・持続酸素投与を要する呼吸器疾患を合併している。

・その他、試験責任医師が本試験への参加が不適切であると判断した患者

**７．プロトコール治療中止基準**

以下の基準のいずれかに該当した場合、プロトコール治療を中止とし、中止理由をカルテおよび症例登録シートに記載する。試験責任医師は被験者に速やかにその旨を通知するとともに、適切な処置を行い、被験者の安全を確保するための検査等を実施し、ｐ７に記載した中止時の有効性評価を行う。以降の治療は規定しない。

1) 理由を問わず入院となった場合

2) 重篤な有害事象(下記)を生じた場合

3) 有害事象が発現し、試験薬の投与継続が困難であると試験責任医師が判断した、または被験者が試験薬の投与中止を希望した場合

4) 被験者の都合により、試験薬の投与継続が困難または試験治療の中止や試験参加辞退の申し出があった場合

5) 登録後に選択基準から逸脱または除外基準に抵触することが判明した場合

6) 被験者の妊娠を確認した場合

7) その他の理由により、医師が試験を中止することが適当と判断した場合

**８．試験期間**

　 各施設での倫理審査会承認後～2019年3月31日（被験者登録）

**９．重篤な有害事象の取り扱い**

重篤な有害事象の定義（薬事法施行規則第273条に準じて定義する）

1）死亡または死亡につながるおそれ

2）治療のための入院または入院期間の延長

3）障害または障害につながるおそれ

4）1)－3)に準じて重篤 5）後世代または先天性の疾病または異常

・試験期間中の全ての重篤な有害事象、試験終了(中止)後に試験との関連性が疑われる重篤な有害事象が発生した場合は直ちに下記まで連絡するとともに、各施設の臨床試験責任者(病院長など)に報告する。

　担当者名：内山　幹

連絡先：TEL 04-7164-1111（内線3201、平日10時～17時）

TEL 04-7164-1111（救急外来，上記以外の時間帯）

**１０．その他の有害事象の評価と報告**

原則として、有害事象および副作用の評価は有害事象共通規準v4.0日本語訳JCOG/JSCO版（CTCAE v4.0）を用いて、有害事象の項目を挙げ、grade判定する。本試験では、CTCAE v4.0で1段階以上gradeが悪化した場合を有害事象と判定する。臨床検査値の異常も同様に判断する。試験開始前より発現していた潰瘍性大腸炎に伴う有害事象(結腸出血、腹痛、下痢、悪心、嘔吐、発熱：下記)についても、gradeが1段階以上悪化した場合に有害事象として症例報告シートにて報告する。試験責任医師は、有害事象の発現を認めた場合、被験者に対して適切な処置を行い、因果関係の有無にかかわらず可能な限り回復するまでその後も追跡観察する。

青黛で予期しない副作用***が発生した場合は，担当医師が保険診療の範囲内で適切な処置を行う。その費用は通常の診療と同様に患者負担となる。

緊急事態が発生した場合は4.9に記した如く，研究代表者にその旨を連絡し、研究代表者は割付け担当者(研究事務局; 筑波大学附属病院　鈴木英雄医師)に当該被験者番号と担当医師名を伝える。割付け担当者は当該症例についてのみ盲検解除を行い、当該症例の担当医師に割付けられた治療法を伝える。重篤な有害事象の定義に該当する場合は試験の継続の可否について研究代表者、及び試験責任医師で協議、決定する。

***下記8項目以外に発生した有害事象を「予期しない副作用」とする．

過去に報告のある有害事象，およびベースラインより悪化した場合に有害事象として扱う潰瘍性大腸炎症状の諸症状（全8項目）

|  | Grade1 | Grade2 | Grade3 | Grade4 | Grade5 |
| --- | --- | --- | --- | --- | --- |
| 頭痛 | 軽度の疼痛 | 中等度の疼痛; 身の回り以外の日常生活動作の制限 | 高度の疼痛; 身の回りの日常生活動作の制限 | - | - |
| 結腸出血 | 軽症; 治療を要さない | 中等度の症状がある;内科的治療または小規模な焼灼術を要する | 輸血/IVRによる処置/内視鏡的処置/待機的外科的処置を要する | 生命を脅かす; 緊急処置を要する | 死亡 |
| 腹痛 | 軽度の疼痛 | 中等度の疼痛; 身の回り以外の日常生活動作の制限 | 高度の疼痛; 身の回りの日常生活動作の制限 | - | - |
| 下痢 | ベースラインと比べて＜4回/日の排便回数増加 | ベースラインと比べて4-6回/日の排便回数増加 | ベースラインと比べて7回以上/日の排便回数増加 | 生命を脅かす; 緊急処置を要する | 死亡 |
| 悪心 | 摂食習慣に影響のない食欲低下 | 顕著な体重減少, 脱水または栄養失調を伴わない経口摂取量の減少 | カロリーや水分の経口摂取が不十分; 経管栄養/TPN/入院を要する | - | - |
| 嘔吐 | 24時間に1-2エピソードの嘔吐（5分以上間隔が開いたものをそれぞれ1エピソードとする） | 24時間に3-5エピソードの嘔吐（5分以上間隔が開いたものをそれぞれ1エピソードとする） | 24時間に6エピソード以上の嘔吐（5分以上間隔が開いたものをそれぞれ1エピソードとする）; TPNまたは入院を要する | 生命を脅かす; 緊急処置を要する | 死亡 |
| 発熱 | 38.0-39.0℃ | ＞39.0-40.0℃ | ＞40.0℃が≦24時間持続 | ＞40.0℃が＞24時間持続 | 死亡 |
| 肺高血圧症 | 極めて軽度の呼吸困難; 理学的/他の検査による所見 | 中等度の呼吸困難, 咳; 心臓カテーテル検査と内科的治療を要する | 低酸素血症や右心不全を伴う高度の症状; 酸素を要する | 生命を脅かす; 緊急処置を要する（例: 気管切開/挿管） | 死亡 |

**１１．試験の終了と早期中止**

**１１．１　試験の終了**

研究事務局は、各研究参加施設からの研究参加人数を一括で管理する．目標人数（68例）に到達した段階でエントリー終了とし，最終エントリー症例の観察期間（2週間）が終了した時点を試験の終了とする．研究事務局は試験が終了した時点で，試験が終了した旨を研究代表者および各医療機関の試験責任医師に速やかに通知する。 試験責任医師は、試験の終了時に、速やかに試験終了報告書を医療機関の長に提出する。

**１１．２　試験の中止、中断**

1) 被験者のリクルートが困難で予定症例を達成することが到底困難であると判断された場合，研究代表者は試験実施継続の可否を検討する。

2) 研究代表者が試験中止の判断をした場合は、本試験を中止し、速やかに各医療機関の試験責任医師、統計解析責任者等に通知する。試験責任医師は、速やかに医療機関の長にその理由とともに文書で報告し、試験中止の決定を行った後、関係者等に速やかに伝達し、中止後の処理にあたるものとする。

3) 試験が早期に中止された場合、速やかに試験結果を公表する。

**１２．被験者の人権（個人情報の保護）に対する配慮**

試験に携わる関係者は、被験者の個人情報およびプライバシー保護に最大限の努力をはらう。試験責任医師は、症例登録シートを提出する際には、被験者識別コード等を用い、医療機関外の者が、被験者を特定できる情報（氏名・住所・電話番号など）は記載しない。

**１３．臨床研究保険**

　本研究で用いる青黛は健康食品として販売されているものであり、医薬品には該当しないため臨床研究保険に加入する必要がない。

**１４．予想される医学上の貢献、および本研究課題の出口**

本研究により、潰瘍性大腸炎に対する青黛の有効性と安全性を確かめることができ、患者に有用な情報を提供できる。なお、本試験の結果は学会報告ならびに論文発表を行う。

**１５．研究に参加した場合に被験者が受ける不利益**

プラセボに割り付けられた際，治療ガイドライン上，本来考慮すべきステロイド製剤の追加など治療適正化されるまでの時間的損失および身体的不利益，服薬状況についてのアンケートを記入する負担、予期せぬ有害事象、補償保険に加入できない研究であることによる身体的・経済的不利益

**１６．患者の費用負担**

本研究で用いる製剤，およびプラセボは共同研究機関である筑波大学薬剤部から各施設に適宜配送する．筑波大学におけるこれらの資金は委任経理金を充てるため，本研究に関する患者負担は生じない．

**１７．研究資金および利益相反**

　青黛の製造・販売を行っているウチダ和漢薬から本研究の結果から製品化を意図した経済的支援はなく，筑波大学の運営交付金（受領者：溝上裕士、寄付者：アストラゼネカ、26年度：研究課題名：潰瘍性大腸炎に対する青黛の有効性と安全性の評価、金額：100万円）を用いるため、同企業との利益相反は生じない。

以上のように，この研究は特定の企業・団体との利害関係はない。研究の実施にあたり、研究の透明性および公平性の確保につとめる。なお、研究者は本学の利益相反管理規程を遵守し、利益相反管理委員会へ手続きを行っている。

**１８．人を対象とする医学系研究に関する倫理指針，ヘルシンキ宣言への対応**

本試験は人を対象とする医学系研究に関する倫理指針、ヘルシンキ宣言を遵守して実施する。

**１９．臨床研究計画の登録**

国立大学附属病院長会議データベース（UMIN）に登録する。

**２０．研究組織**

２０．０　プロトコール作成

　筑波大学附属病院　光学医療診療部　鈴木英雄

２０．１　研究代表者

東京慈恵会医科大学附属柏病院 消化器・肝臓内科 内山幹

２０．２　参加医療機関（試験実施場所）と試験責任医師

1, 筑波大学附属病院　光学医療診療部　鈴木英雄、溝上裕士

2, 東葛辻仲病院 胃腸科 望月暁

3, 東京医科大学茨城医療センター　消化器内科　岩本淳一

4, 大森敏秀胃腸科クリニック　消化器内科　大森敏秀

5, 小山記念病院　内視鏡センター　若山真理子

6, おなかクリニック　院長　村井隆三，IBD外来　内山幹

7, 日製日立総合病院　柿木信重，谷中昭典

8，日製ひたちなか総合病院　廣島良規

9, 水戸済生会病院　柏村浩

10, 船橋診療所　笠貫順二

11, 東京女子医科大学八千代医療センター　消化器内科　白戸泉

12, 谷津保険病院　消化器内科　星野容子

13, 龍ヶ崎済生会病院　間宮孝

14, 千葉西総合病院　消化器内科 梅木清孝

15, 医療法人社団寿和会　白戸胃腸科・外科　白戸泉

16, 独立行政法人国立病院機構　霞ヶ浦医療センター　廣瀬充明

**２１.　研究事務局**

　筑波大学附属病院　光学医療診療部　鈴木英雄

連絡先：TEL 029-853-3218（消化器内科医局、平日10時～17時）

TEL 029-853-3110（救急外来，上記以外の時間帯

E-mail : hideoszk@md.tsukuba.ac.jp

**２２. 実施計画書等の変更**

実施計画書や同意説明文書の変更（改訂）を行う場合は予め倫理審査委員会の承認を必要とする。

**２３. 緊急連絡先または相談窓口**

病院名　：　東京慈恵会医科大学附属柏病院

科名・医師名：　消化器･肝臓内科，内山　幹（うちやま　かん）

連絡先　：　TEL：04-7164-1111　(内線3201)

　　対応時間： 平日・土曜日　午前9時～午後5時00分

附属柏病院個人情報保護相談窓口

連絡先　：　TEL: 04-7164-1111　（内線2183）

　　　　（休日を除く午前9時～午後5時）

参考文献

1. Fukunaga K, Ohda Y, Hida N, Iimuro M, Yokoyama Y, Kamikozuru K, Nagase K, Nakamura S, Miwa H, Matsumoto T. Placebo controlled evaluation of Xilei San, a herbal preparation in patients with intractable ulcerative proctitis. J Gastroenterol Hepatol. 2012 Dec;27(12):1808-15.

2. Suzuki H, Kaneko T, Mizokami Y, Narasaka T, Endo S, Matsui H, Yanaka A, Hirayama A, Hyodo I. Therapeutic efficacy of the Qing Dai in patients with intractable ulcerative colitis. World J Gastroenterol. 2013 May 7;19(17):2718-22.
