# Supplementary material for: Efficacy and safety of short-term therapy with indigo naturalis for ulcerative colitis: An investigator-initiated multicenter double-blind clinical trial
Source: PLoS One. 2020 Nov 5;15(11):e0241337. doi: 10.1371/journal.pone.0241337 (PMC7644062; doi:10.1371/journal.pone.0241337)
Supplement: S2 File — (DOCX) [file pone.0241337.s003.docx]

Evaluation of efficacy and safety of the Qing Dai (Indigo naturalis) in patients with ulcerative colitis: A multicenter, double-blind, randomized controlled trial.

Research protocol

Ver.1-11

principal investigator

Kan Uchiyama

Division of Gastroenterology and Hepatology, The Jikei University Kashiwa Hospital

Collaborator

Toshifumi Ohkusa:Division of Gastroenterology and Hepatology, The Jikei University Kashiwa Hospital

Shunichi Odahara:Division of Gastroenterology and Hepatology, The Jikei University Kashiwa Hospital

Zensho Ito:Division of Gastroenterology and Hepatology, The Jikei University Kashiwa Hospital

Shinichiro Takami:Division of Gastroenterology and Hepatology, The Jikei University Kashiwa Hospital

Expected Clinical Trial Period：After Institutional Review Board approval to March/2019

Contents

1. Background and aims
2. Subjects
3. How to explain to the subject and obtain consent
4. Methods and evaluation
5. Eligible criteria
6. Exclusion criteria
7. Protocol discontinuation criteria
8. Research period
9. Handling about serious adverse event
10. Evaluation and reporting of other adverse events
11. Termination
12. Consideration for the human rights of subjects (protection of personal information)
13. Clinical research insurance
14. Anticipated medical contributions and outcomes
15. Disadvantage to subjects
16. Patient cost sharing
17. Research funding and conflicts of interest
18. Ethical guidelines for medical research involving human subjects, response to the Declaration of Helsinki
19. Clinical research protocol registration
20. Organization
21. Research office
22. Implementation plan revision
23. Contact information

**１．Background and aims**

Ulcerative colitis (UC) is an intractable inflammatory bowel disease that is rapidly increasing with the westernization of our diets. Traditionally, 5-aminosalicylic acid (5-ASA), steroids, immunomodulators (azathioprine, 6-MP) and other drugs and cytoapheresis (L-CAP, G-CAP) have been used for UC, but the recent introduction of anti-TNF-alpha antibody (infliximab, adalimumab) and calcineurin inhibitors (tacrolimus) has led to a wide range of treatment options. On the other hand, certain health products have also become widespread in some patients, but the reality of these products is not clear.

The treatment of UC is based on disease activity indices with clinical symptoms such as stool frequency and blood in the stool (Lichtiger index) and endoscopic activity (Mayo subscore). In the guideline for the treatment of ulcerative colitis (revised March 2014, Japanese "Investigation of intractable intestinal disorders" group), 5-ASA is used as a basic drug and a steroid, cytoapheresis and immunomodulator should be used for patients who did not respond to 5-ASA, but standardization of treatment has not been established. It is likely that health products are used under these treatments, but there are no reports of their effects on the course of the disease.

The Qing Dai, also called as Indigo naturalis (IN), is a blue pigment found in leaves and stems of plants such as Assam indigo, false indigo and woad, it is available as health products. On the other hand, the enema of the Chinese herbal medicine Xileisan, which is mainly composed of IN, has been used for the treatment of ulcerative colitis in China, and the efficacy of suppositories against placebos has been reported in Japan. In recent years, it has also been reported that oral administration of IN was effective in several UC patients, but scientific evidence is lacking because of the limited case reports.

In this study, we evaluate the efficacy and safety of IN in a multicenter, placebo-controlled, double-blind study to obtain higher scientific evidence to conclude the efficacy and safety of IN.

**２．Subjects**

A total of 68 patients* with mild to moderate disease activity who are refractory to or unresponsive to existing treatment and who are attending the Inflammatory bowel disease (IBD) outpatient clinic at each institution. Because UC is a disease that predominantly affects children and adolescents, this study is conducted in children and adolescents over 16 years of age. Patients with over 16 years old and under 20 years old requires the consent from their parents or guardians. Pediatric patients (younger than 16 years old) and those aged between 16 and 20 years old who do not have guardian or patient’s consent are not included.

＊If the efficacy rate of the IN group is 70%, the efficacy rate of the placebo group is 30%, alpha error 0.05, and power 0.8, then the required number of cases is 58 cases in total. The required number of patients is 29 in each group. The number of patients who dropped out of the study is estimated to be 5 in each group and 34 in each group, for a total of 68.

**３. How to explain to the subject and obtain consent**

**３．１　Obtaining Consent**

Prior to implementation of the study, the principal investigator or collaborator will obtain the patient's consent to participate in the study by means of a consent document that includes the following items, which is sufficiently explained to the patient to confirm that he or she has fully understood the contents of the document. In that time, the patient has an opportunity to ask questions and enough time to decide whether or not to cooperate with the study. The consent form should be signed and dated by the study investigator who provided the explanation and by the patient. If the patient is a minor, as a rule, the parents should be selected as a surrogate to obtain informed consent. However, if the patient is between 16 and 20 years old, the consent should be obtained in addition to the above surrogate. Participation in this study is voluntary and patients will not be disadvantaged if they do not participate in this study. And even if patients agree to participate, they can withdraw this at any time.

1. The purpose of the study
2. The methods
3. The expected period of participation
4. The number of patients expected to participate
5. The examinations associated with the study
6. Expected effects, side effects, and abnormal clinical laboratory values
7. Other possible treatment options
8. The possibility that the results of this study may be opened to public with personal privacy protected.
9. The cost of treatment in this study
10. Compensation for health hazard
11. On being informed of any new information as soon as it becomes available
12. Handling of questionnaires and data during and after study
13. Inquiries and contact information

**３．２　Time to obtain consent**

Written consent must be obtained before registration.

**４．Methods and evaluation**

**４．１ Randomization and case enrollment**

　　Case enrollment is done using HOPE eACReSS, a clinical research support system. This system allows for web-based patient registration and randomization. Using this system, a randomized grouping is performed between the IN and placebo groups. Patient information, pre- and post-dose disease activity index (Lichtiger index), endoscopic findings, examination results, and the presence or absence of side effects can be entered.

＜Lichtiger Index＞

| **Symptom** | **Score** |
| --- | --- |
| *Diarrhea (no. of daily stools)* |  |
| 0–2 | 0 |
| 3 or 4 | 1 |
| 5 or 6 | 2 |
| 7–9 | 3 |
| 10 | 4 |
| *Nocturnal diarrhea* |  |
| No | 0 |
| Yes | 1 |
| *Visible blood* | (% of movements) |
| 0 | 0 |
| Less than 50 | 1 |
| Greater than 50 | 2 |
| 100 | 3 |
| *Fecal incontinence* |  |
| No | 0 |
| Yes | 1 |
| *Abdominal pain or cramping* |  |
| None | 0 |
| Mild | 1 |
| Moderate | 2 |
| Severe | 3 |
| *General well being* |  |
| Perfect | 0 |
| Very good | 1 |
| Good | 2 |
| Average | 3 |
| Poor | 4 |
| Terrible | 5 |
| *Abdominal tenderness* |  |
| None | 0 |
| Mild and localized | 1 |
| Mild to moderate and diffuse | 2 |
| Severe or rebound | 3 |
| *Need for antidiarrhea drugs* |  |
| No | 0 |
| Yes | 1 |

**４．２　Preparation and management of IN or placebos**

　　The IN and placebo are packed into 1 capsule, 100mg each, at Uchida Wakanyaku. Placebo is rice starch. The IN and placebos are kept in an airtight container to avoid high temperature and humidity. These will be packaged together with the dehumidifier in plastic bottles as formulation A or formulation B and delivered to each facility. The product should be kept away from high temperatures and high humidity and should not be used after its expiration date.

　　The registration number on the web registry should be written on the cap of the plastic bottle with an oil-based marker when handing the assigned formulation to the patient.

**４．３　Dosage**

　　In addition to regular medication (e.g. 5-ASA), the patients should also take a IN or placebo 5 capsules (500 mg), twice daily for 2 weeks.

**４．４　Questionnaire**

　The subjects are required to write their dose status, number of bowel movements, stool characteristics, and degree of bleeding in printed questionnaire.

**４．５　The end of the study**

The investigator enters clinical and endoscopic findings, test results, and adverse events after 2 weeks. If it is cancelled, also state the reason for the cancellation. At the end of the period, the excess product should be collected and stored at each facility until unblinded.

| Implementation items | | Period | | Cancellation |
| --- | --- | --- | --- | --- |
|  |  | Start date（pre） | Two weeks after**^＊＊^** |  |
| Selection/exclusion criteria /obtaining consent | | ● | － | － |
| Subject's background and pretreatment status | | ● | － | － |
| Side effects | | － | ● | ● |
| 評  価 | Clinical activity index (Lichtiger index) | ● | ● | ● |
|  | Blood examination**^＊＊＊^** | ● | ● | ● |
|  | Endoscopy**^＊^** | （●）  (Within 2 weeks from the start) | （●）  (Within 1 week from the end) | － |
|  | Patient Record | － | ● | ● |
|  | Fecal examination******** | （●） | （●） |  |

**＊**Arbitrary. Observation of the rectum only by enema is also available. Biopsies are not required.

**＊＊**If the patient wishes to continue taking the product, purchase IN on their own as a health food and take it after reporting to their investigators.

**＊＊＊**Complete blood count, total protein, albumin, CRP, and erythrocyte sedimentation rate are required for blood examination.

**＊＊＊＊**In the case of endoscopy, the collection of intestinal fluid may be used as a substitute.

**４．６　Primary Endpoint**

　　Percentage of improvement in the clinical activity index (Lichtiger index) of 50% or more after 2 weeks of treatment*

*An "improvement" is defined as a score reduction of 50% or more of the baseline.

**４．７　Secondary Endpoint**

Proportion of subjects with a reduction of one or more in endoscopic findings (Endoscopic Mayo score) after treatment **

**A score reduction of 1 or more is defined as an "improvement".

Percentage of subjects experiencing new adverse physical events (headache, diarrhea, shortness of breath, fever, etc.) during treatment

Proportion of subjects with new abnormal blood examination results during the treatment period and their details

Changes in gut microbiota before and after administration

**４．８　Blind Search Disengagement**

Unblinding is completed after the data is fixed except in cases of emergency when the study is performed in accordance with the 4.9 procedure.

**４．９　Emergency key management and unblinding procedures**

Emergency keys is kept by the assignment coding staff and will not be opened except in case of an emergency. An emergency is defined as when an adverse event or serious adverse event occurs, with different measures depending on whether the allocation is IN or not, and when it meets the definition of a serious adverse event in Chapter 9. In the case of an emergency, the principal investigator needs to notify the representative of each institution, and inform the person in charge of the assignment (research office; University of Tsukuba Hospital, Dr. Hideo Suzuki) of the subject's number and the name of the investigator in charge. The allocator unblind only the case and inform the physician in charge of the case of the allocated treatment. If the case meets the definition of a serious adverse event, the principal investigator and other investigators discuss and decide whether to continue the study. No unblinding is done to determine the post-treatment method.

**４．１０　Methods of Statistical Analysis**

　　　The percentage of subjects who improved the clinical activity index by more than 50% between the IN group and the placebo group is compared using the Chi-square test, and the change in the above index is evaluated using the Wilcoxon test. When the efficacy of the IN group is confirmed, multivariate analysis (logistic regression analysis) is performed using various patient background items such as disease duration and disease type to examine effective predictive factors.

**４．１１　Monitoring**

　　　Monitoring is specified separately in monitoring protocol.

**５．Eligible criteria**

　・16years and older

　・Performance status (Eastern Cooperative Oncology Group (ECOG)) of 0 or 1

・Ulcerative colitis with mild to moderate activity (Lichtiger index 5-10)

・Outpatient visit

　・Tolerant or refractory to existing treatments (Any drug dosage, including relapse during dose reduction)

・Blood examination within 2 weeks need to meet all of the following conditions

i) Hemoglobin (Hb)≧9g/dl

ii) Aspartate aminotransferase (AST), alanine aminotransferase (ALT)≦facility standard

iii) Serum creatinine≦facility standard

・Written consent need to be obtained from the subjects to participate in this study

**６．Exclusion criteria**

　・newly start or dose-up of 5-ASA within two weeks

　・newly start or dose-up of steroids within two weeks

　・newly start or dose-up of immunomodulators, such as azathioprine/6-methyl mercaptopurine within three months

　・newly start of cytoapheresis within three months

　・newly start or dose-up of biologics (Infliximab or Adalimumab) or tacrolimus within three months

　・any herbal medicine use containing IN

　・a possibility of pregnancy, during pregnancy, or lactating

・any mental illness that interfere to participate this study

・active bacterial/fungal infections (A fever of 38.5°C or higher, with evidence of bacterial infection on diagnostic imaging or bacteriological tests)

・history of myocardial infarction or unstable angina within three months

・uncontrollable hypertension

・respiratory disease requiring a sustained oxygen administration

・Others whose participation in the study is deemed inappropriate by the investigator

**７．Protocol discontinuation criteria**

If any of the following criteria are met, the protocol treatment is discontinued and the reason for discontinuation is noted in the medical record and case registration sheet. The principal investigator promptly notifies the subject, take appropriate action, perform examinations and other measures to ensure subject safety, and evaluate the efficacy of the study at the time of discontinuation as described. No further treatment is prescribed.

1) When the subject is hospitalized for any reason

2) When a serious adverse event (see below) occurs

3) When an adverse event occurs and the principal investigator determines that continuation of the study is not feasible, or the subject wishes to discontinue.

4) When the subject is unable to continue receiving the products, or wish to discontinues the study due to the subject's circumstances

5) If, after registration, the subjects are found to have deviated from the selection criteria or violated the exclusion criteria

6) In case of subject's pregnancy

7) When the investigator deems to stop the study for any other reason

**８．Research period**

　 After each institutional review board approval to 3/31/2019 (Subject registration)

**９．Handling about serious adverse event**

Definition of serious adverse events (Defined in accordance with Article 273 of the Japanese Pharmaceutical Affairs Law Enforcement Regulations)

1）May result in death or fatalities

2）Hospitalization or extended hospitalization for treatment

3）Impairment or threat of impairment

4）Serious conditions in accordance with 1)-3)

5）Subsequent or congenital diseases or abnormalities

・All serious adverse events during the study period, and any serious adverse events after the completion (discontinuation) that are suspected to be related to the study, should be reported immediately to the following and to the person in charge (e.g., hospital director) at each site

　Name of person in charge：Kan Uchiyama

Contact：TEL 04-7164-1111 (Int. 3201、weekday 10AM-5PM)

TEL 04-7164-1111 (emergency office，Times other than the above)

**１０．Evaluation and reporting of other adverse events**

In principle, the evaluation of adverse events and side effects is based on the Common Terminology Criteria for Adverse Events v4.0 (CTCAE v4.0) JCOG/JSCO version, list and grade the items for adverse events. In this study, a grade worsening of one or more levels in CTCAE v4.0 is considered as an adverse event. Abnormal laboratory values are determined in the same way. Adverse events associated with ulcerative colitis (colonic bleeding, abdominal pain, diarrhea, nausea, vomiting, and fever: see below) is reported on the case report sheet if the grade worsens by one or more levels. If an adverse event is observed, the study investigator takes appropriate treatment to the subject and follow him/her thereafter until he/she recovers to the best of his/her ability, with or without a causal relationship. When an unexpected side effect*** occurs with IN, investigator needs to take appropriate measures within the scope of insurance treatment. The cost is borne by the patient as well as the usual practice. In the case of an emergency, the investigator needs to notify to the principal investigator of the emergency as described in 4.9, and the principal investigator send the subject number and name of the investigator to the charge of the allocation (research office; University of Tsukuba Hospital, Dr. Hideo Suzuki). The allocator unblind the case and inform the investigator in charge about the allocated treatment. If the case meets the definition of a serious adverse event, the principal investigator and investigators discuss and decide whether to continue the study.

*** Adverse events that occur in addition to the following eight items are referred to as "unexpected side effects”.

Symptoms of ulcerative colitis symptoms reported in the past and treated as an adverse event if they are worse than the baseline（Total 8 items）

|  | Grade1 | Grade2 | Grade3 | Grade4 | Grade5 |
| --- | --- | --- | --- | --- | --- |
| Headache | Mild pain | Moderate pain; limiting instrumental ADL | Severe pain; limiting self-care ADL | - | - |
| Colonic hemorrhage | Mild; intervention not indicated | Moderate symptoms; medical intervention or minor cauterization indicated | Transfusion, radiologic, endoscopic, or elective operative intervention indicated | Life-threatening consequences; urgent intervention indicated | Death |
| Abdominal pain | Mild pain | Moderate pain; limiting instrumental ADL | Severe pain; limiting self-care ADL | - | - |
| Diarrhea | Increase of <4 stools per day over baseline; mild increase in ostomy output compared to baseline | Increase of 4 - 6 stools per day over baseline; moderate increase in ostomy output compared to baseline | Increase of >=7 stools per day over baseline; incontinence; hospitalization indicated; severe increase in ostomy output compared to baseline; limiting self-care ADL | Life-threatening consequences; urgent intervention indicated | Death |
| Nausea | Loss of appetite without alteration in eating habits | Oral intake decreased without significant weight loss, dehydration or malnutrition | Inadequate oral caloric or fluid intake; tube feeding, TPN, or hospitalization indicated | - | - |
| Vomiting | 1 - 2 episodes (separated by 5 minutes) in 24 hrs. | 3 - 5 episodes (separated by 5 minutes) in 24 hrs. | >=6 episodes (separated by 5 minutes) in 24 hrs.; tube feeding, TPN or hospitalization indicated | Life-threatening consequences; urgent intervention indicated | Death |
| Fever | 38.0-39.0℃ | ＞39.0-40.0℃ | >40.0 ℃for <=24 hrs. | >40.0℃ for >24 hrs. | Death |
| Pulmonary hypertension | Minimal dyspnea; findings on physical exam or other evaluation | Moderate dyspnea, cough; requiring evaluation by cardiac catheterization and medical intervention | Severe symptoms, associated with hypoxemia, right heart failure; oxygen indicated | Life-threatening airway consequences; urgent intervention indicated (e.g., tracheotomy or intubation) | Death |

**１１．Termination**

**１１．１　End of study**

The research office manages the number of participants from each participating institution. Entry is terminated when the total number of patients is reached, and the study is terminated at the end of the 2-week observation period of the last entry. Upon completion of the study, the research office promptly notifies the principal investigator and other investigators in each institution that the study has been completed. The investigators submit a report on the completion of the study to the representative of each institution as soon as possible at the end of the study.

**１１．２　Cancellation or suspension of the study**

1) The principal investigator considers whether or not to continue the study if it is determined that recruitment of subjects is difficult and there is little or no chance of achieving the planned patient population.

2) When the principal investigator decides to discontinue, the principal investigator notifies to the other investigators and statistical analysis immediately. The principal investigator promptly reports in writing to the representative of the institution with the reasons for the discontinuation and promptly communicate to the other investigators about discontinuation to take care of the further treatment.

3) In the event of early termination of the study, the results will be made public as soon as possible.

**１２．Consideration for the human rights of subjects (protection of personal information)**

All parties involved in the study make every effort to protect the personal information and privacy of subjects. When submitting the case enrollment sheet, the investigator uses a subject identification code and do not include any personal information (e.g., name, address, phone number, etc.) that would allow to identify the subject by outside of the institution.

**１３．Clinical research insurance**

Because IN used in this study are sold as a health food and do not fall under the category of pharmaceutical products, it is not necessary to join the clinical research insurance.

**１４．Anticipated medical contributions and outcomes**

Through this study, we can confirm the efficacy and safety of IN for ulcerative colitis and provide useful information to patients. The results of this study will be reported at the conference and presented in a paper.

**１５．Disadvantage to subjects**

In the case of being assigned to placebo group, time loss and physical disadvantages until appropriate treatment such as addition of steroids that is considered in the treatment guidelines; the burden of completing questionnaires about medication status; unexpected adverse events; and physical and financial disadvantages of being in a study with no coverage.

**１６．Patient cost sharing**

The IN and placebos used in this study will be delivered from University of Tsukuba to each facility in appropriate methods. Because the University of Tsukuba uses delegated funds, no patient costs will be incurred for this study.

**１７．Research funding and conflicts of interest**

　Because there was no financial support from Uchida Wakanyaku, the manufacturer and seller of IN, and delegated funds from the University of Tsukuba is used, there is no conflict of interest with that company.

As mentioned above, this study has no conflict of interest with any particular company or organization. Efforts will be made to ensure transparency and fairness in this research.

Furthermore, the researcher complies with the Conflict of Interest Management Regulations of the University and has taken the necessary procedures to the Conflict of Interest Management Committee.

**１８．Ethical guidelines for medical research involving human subjects, response to the Declaration of Helsinki**

The study is conducted in compliance with the ethical guidelines for medical research involving human subjects and the Declaration of Helsinki.

**１９．Clinical research protocol registration**

This study is registered to the University Hospital Medical Information Network database (UMIN).

**２０．Organization**

２０．０　Protocol development

　　Hideo Suzuki: University of Tsukuba

２０．１　Principal investigator

Kan Uchiyama:The Jikei University Kashiwa Hospital

２０．２　Participating medical institutions (study location) and study investigators

1, Hideo Suzuki,Yuji Mizokami:University of Tsukuba

2, Satoshi Mochizuki:Tokatsu Tsujinaka Hospital

3, Junichi Iwamoto:Tokyo Medical University Ibaraki Medical Center

4, Ohmori Toshihide:Ohmori Toshihide Gastrointestinal Clinic

5, Mariko Wakayama:Koyama Memorial Hospital

6, Ryuzo Murai,Kan Uchiyama:Onaka Clinic

7, Nobushige Kakinoki, Akinori Yanaka:Hitachi General Hospital

8，Yoshinori Hiroshima:Hitachinaka General Hospital

9, Hiroshi Kashimura:Mito Saiseikai General Hospital

10, Junji Kasanuki:Funabashi Minato Clinic

11, Izumi Shirato:Tokyo Women's Medical University Yachiyo Medical Center

12, Yoko Hoshino:Yatsu Hoken Hospital

13, Takashi Mamiya:Ryugasaki Saiseikai Hospital

14, Kiyotaka Umeki:Chiba-Nishi General Hospital

15, Izumi Shirato:Shirato Ichouka Geka

16, Mitsuaki Hirose:National Hospital Organization Kasumigaura Medical Center

**２１．Research office**

　Hideo Suzuki:University of Tsukuba Hospital

TEL +81-29-853-3218（Department Office, Weekday 10AM-5PM）

TEL +81-29-853-3110（Emergency Office，Times other than the above）

E-mail : hideoszk@md.tsukuba.ac.jp

**２２. Implementation plan revision**

Any changes (revisions) to the implementation plan or consent document require prior approval of the Institutional Review Committee.

**２３. Contact information**

The Jikei University Kashiwa Hospital

Division of Gastroenterology and Hepatology，Kan Uchiyama

TEL：+81-4-7164-1111　(Int. 3201) Weekday & Saturday　9AM-5PM

Personal Information Protection Consultation

TEL: +81-4-7164-1111　（Int. 2183）（9AM-5PM, excluding holidays）

Reference

1. Fukunaga K, Ohda Y, Hida N, Iimuro M, Yokoyama Y, Kamikozuru K, Nagase K, Nakamura S, Miwa H, Matsumoto T. Placebo controlled evaluation of Xilei San, a herbal preparation in patients with intractable ulcerative proctitis. J Gastroenterol Hepatol. 2012 Dec;27(12):1808-15.

2. Suzuki H, Kaneko T, Mizokami Y, Narasaka T, Endo S, Matsui H, Yanaka A, Hirayama A, Hyodo I. Therapeutic efficacy of the Qing Dai in patients with intractable ulcerative colitis. World J Gastroenterol. 2013 May 7;19(17):2718-22.
