# Supplementary material for: Efficacy and safety of short-term therapy with indigo naturalis for ulcerative colitis: An investigator-initiated multicenter double-blind clinical trial
Source: PLoS One. 2020 Nov 5;15(11):e0241337. doi: 10.1371/journal.pone.0241337 (PMC7644062; doi:10.1371/journal.pone.0241337)
Supplement: Attachment — Submitted filename: Responce to reviewers.docx [file pone.0241337.s004.docx]

Dear Professor Hepp,

Thank you very much for reviewing our manuscript and offering valuable advice.

We have addressed comments with point-by-point responses and revised the manuscript accordingly.

**Journal Requirements:**

1. Please ensure that your manuscript meets PLOS ONE's style requirements, including those for file naming.

We rewrote the manuscript to PLOS ONE's style.

2. Please include a sample size and power calculation in your methods section.

We included a sample size and power calculation in methods section.

**Response to Reviewer**

We wish to express our appreciation to the reviewer for the insightful comments, which have helped us significantly improve the paper. The followings are the responses for reviewers.

Reviewer #1:

1) Lack of repetitive testing.

We recognize the importance of repetitive testing. As mentioned in the Methods section, repetitive testing was not possible in this study because of the time constraint until Clinical Research Act execution.

2) The sample size of patients is small.

If it was assumed that the efficacy of IN was 80% and that of placebo was 40% with α = 0.05, β = 0.2 and power = 0.8, the number of samples was calculated to 22 cases in each group. Furthermore, similar previous studies have found results with a sample size of around 20, and we do not believe that our sample size is extremely small. As mentioned above, we included a sample size and power calculation in methods section.

3) The administration time is short, which cannot explain the long-term efficacy or safety.

Since long-term treatment is associated with increased risk of pulmonary arterial hypertension (PAH) or intussusception, the efficacy and safety of IN were evaluated in this study in a short period. The risk of PAH or intussusception in long-term treatment was added to the introduction.

4) Endoscopic evaluation cannot be performed in a short period of time.

Because the Lichtiger index and endoscopic severity have been reported to have strong correlation, the endoscopy was not required in this study. This was added to the discussion section.

Reviewer #2:

Introduction

There has been already published several academic researches about IN in the treatment for UC. The traditional use of IN should be short (or may not be needed).

Since the pulmonary arterial hypertension (PAH) or intussusception have been reported in long-term treatment of IN, a short period use was evaluated in this study. The risk of PAH or intussusception in long-term treatment and anticipated benefit of short-term use were added in the introduction.

Result

1.They showed baseline characteristics and said there were no significant difference between two group. How about endoscopic score?

Endoscopy was not required in this study, so we did not compare endoscopy scores. The Lichtiger index and endoscopic severity have been reported to have strong correlation and it was added to the discussion section.

2.They described prior treatment. I am interested to see if they could stop these treatments after IN treatment.

Due to protocol restrictions, the dose of the prior treatments could not be reduced during the trial period of 2 weeks, but some patients who continued the IN voluntarily could reduce or discontinue the prior treatments such as enema preparations. It is difficult to describe these cases as a result because of the lack of tracking.

3.Pulmonary arterial hypertension is serious adverse event. So, they should pay attention for PAH. Did they monitor something in the patients to find adverse event early?

Yes. We monitored shortness of breath which was associated with PAH carefully during the trial period. This was added in Materials and methods. For patients who continue to take it voluntarily, we are trying to measure brain natriuretic peptide (BNP) regularly in order to detect PAH early. This was added in the Discussion section.

Discussion

1.Given the mechanism of IN they described, is IN useful for other IBD?

For unknown reasons, it was reported that the effect on Crohn’s disease was not as high as on UC. This report was added in discussion.

2.I understand main outcome of this study is 2 weeks data, however, usually data (even at 24 week) should described in result section. Reader will be interest in the results at 24 weeks.

The results after 24 weeks are shown in Supplement Fig 1. These results at week 24 are outside the protocol of this study, therefore, we mentioned in the discussion section. According these results, 30 of 32 patients remained remission.

Are (1) the funding sources, and (2) the role of the funder(s) described?

I could not find the sentences about funding. If there was funding, please described it.

We have no funding interests and it is declared at the last line in Acknowledgement.

Again, thank you for giving us the opportunity to strengthen our manuscript with your valuable comments and queries. We hope that the revised manuscript is suitable for publication.

Sincerely,

Kan Uchiyama

Shinichiro Takami

Hideo Suzuki
