# Supplementary material for: Efficacy and safety of short-term therapy with indigo naturalis for ulcerative colitis: An investigator-initiated multicenter double-blind clinical trial
Source: PLoS One. 2020 Nov 5;15(11):e0241337. doi: 10.1371/journal.pone.0241337 (PMC7644062; doi:10.1371/journal.pone.0241337)
Supplement: Attachment — Submitted filename: Responce to reviewers2.docx [file pone.0241337.s005.docx]

Professor Chuang,

I appreciate valuable comment and suggestion.

We have addressed comments with point-by-point responses and revised the manuscript. The followings are the answers for reviewers.

Response to Reviewer

Reviewer #1:

1, The short duration of administration could not indicate long-term efficacy or safety. Lack of long-term patient with follow-up is unable to identify whether the patient has recurrence or not. For the conventional patients with UC, the short-term treatment with IN is of little significance. In contrast, more studies currently focus on the maintenance treatment with IN. In addition, many scholars have studied IN for the treatment-refractory patients with UC. In conclusion, research into short-term therapy with IN is less innovative.

2, The IN clinical trials for the treatment of UC, relatively complete is Naganuma et al.published in Gastroenterology the Efficacy of Indigo naturalis in a Multicenter Randomized Controlled Trial of Patients with Ulcerative Colitis. What is the innovation of your trial compared with Naganuma et al. and other clinical trials.

3, However, IN should not yet be used because of the potential for adverse effects, including pulmonary arterial hypertension or intussusception, and this could be the main reason for that China has not yet used IN for UC patients in large-scale. And no serious adverse events were observed after short-term administration for 2 weeks as mentioned in the author’s manuscript. Whether this could be an advantage as a short-term treatment is worth exploring. It may be significant to study the optimal treatment course and dosage of IN for UC on the premise of minimizing the occurrence of severe adverse reactions.

This study is the first to report short-term administration of IN, indicating the potential to avoid serious side effects including PHT. Naganuma et al. administered IN for eight weeks, but we showed similar efficacy only for two weeks. As prednisolone or tacrolimus have been used for remission induction but not maintenance, we believe it is innovative that IN could be used in the same strategies. These descriptions are added in the “Discussion”.

Reviewer #2:

I apologize for your misunderstanding. I mentioned last time that the description in the introduction section is not academic. There have already been many academic studies about IN. The descriptions about "the plant", “kai Ban Ben Cao” and “Furikake” were not related to this study. Please shorten the description of traditional use of IN.

We have shorten the description of traditional use of IN in the “Introduction”.

“the endoscopy was not required” added in the revise is not logical. The endoscopy was not required in the “study design” (especially at 2 weeks). This paper described information at entry, 2 weeks, and 24 weeks. Please write a suppressed expression instead of “the endoscopy was not required”.

The “the endoscopy was not required” was deleted in discussion and suppressed expression “Due to the short administration period of 2 weeks, endoscopic observation before and after was not mandatory” was added in study design.

Reviewer #3:

1) The results after 24 weeks are of interest. However, they are not presented properly in the paper. Since they are somewhat observational, it is important to clearly describe why and how they were obtained, as well as the results and their potential interpretation. Currently, these pieces of information are all located in the Discussion section, which is incorrect.

Please provide:

* A clear description of the rationale for additional assessment in the Background section (i.e., Lines 235-243),

* A clear description of methods of data collection and data actually collected in the Methods section (i.e., Lines 244-247)

* The results in the Results section (i.e., Supplementary Figure 1 and Lines 247-256), and

* Interpretation in the Discussion section (i.e., Lines 256-264).

We added some sentences and made revisions in each section according to the reviewer’s suggestions.

* Supplementary Figure 1 should then be promoted to "Figure" status.

Supplementary Figure 1 was changed to Figure 5.

2) In general, the statistical analysis is serviceable. One can argue endlessly about whether the authors ought to perform an analysis of covariance or a repeated measures analysis, but it seems very unlikely to change the key results. Additionally, the raw data for Lichtiger index are shown, so the reader may judge.

Repeated measures analysis is used to compare data at three or more time points, but in this study we used the paired t-test because it is two points before and after.

3) The discussion of sample size is moot at this point, since the study is complete and sample size is now determined. Naturally, it would be better to have more patients on study, but we must do what we can. The authors' response on this issue is satisfactory.

Thank you for your comment. There is no correction in this regard.

4) Although it gets the job done, the analysis presented in Lines 181-184 is somewhat crude. Table 3 should probably be demoted to "Supplementary Table" status.

The authors do not need to amend the analysis. However, they may wish to perform simple linear modelling of the Lichtiger index on these covariates instead of dichotomizing the data. This method should provide more power to detect associations. If not already done, the authors should definitely plot the Lichtiger index across each of these covariates to screen for patterns.

Table 3 was demoted to "Supplementary Table 3".

Although the Lichtiger index across each of these covariates to screen for patterns is important to see the correlations between each item and the degree of response, we wanted to clarify the characteristics of the group with the higher response, i.e., the group with the great effect, so we performed a comparison between the two groups.

*** The following are minor suggestions: ***

* Line 68: Change to "or for intolerant patients".

We changed to "or for intolerant patients".

* Line 110: In this case, does "intervention" refer to "any intervention" or "any UC intervention"?

We changed to "any UC intervention".

* Lines 119-120: Change to "This system was used to perform minimization randomization using the Lichtiger index before and after administration, [SPECIFY BLOOD TEST DATA USED], and presence or absence of adverse events [SPECIFY SPECIFICS OF ADVERSE EVENTS THAT WERE USED]." Then, probably Lines 121-125 may be deleted.

There were some difficulties in the original content, so we have revised it based on your instructions.

* Line 145-146: Change to "The effect of treatment on response rate was assessed using the chi-square test. The differences in Lichtiger index between groups was assessed using the unpaired two-sided t-test, and the difference within groups over time was assessed using the paired two-sided t-test. Statistical significance was declared at the 0.05 level." Please check that t-tests were performed using the two-sided version.

* In Line 214, the authors describe a "dose-dependent linear trend". This seems a bit of a stretch, since the data given seem to describe a sharp rise and then a plateau instead. I suggest to amend this to a "dose-dependent response".

* Line 230: Change to "and it is possible that the exogenous".

* Please harmonize the use of colors, labels, and the placement of legends (bottom or right) among the figures.

We corrected them as instructed.

Hideo Suzuki
